# Retraction: Overweight and Cognitive Performance: High Body Mass Index Is Associated With Impairment in Reactive Control During Task Switching

**DOI:** 10.3389/fnut.2020.615640

**Published:** 2020-11-11

**Authors:** Frontiers Editorial Office

The journal hereby retracts the above cited article. This follows the recommendations of an investigation by Leiden University's Committee on Scientific Integrity which found that the paper contained gross data manipulation. These issues could not have been detected during review but nevertheless invalidate the study's findings. The retraction has been approved by the journal Chief Editors.

